# Optical Resolution Photoacoustic Microscopy of Ovary and Fallopian Tube

**DOI:** 10.1038/s41598-019-50743-7

**Published:** 2019-10-04

**Authors:** Bin Rao, Xiandong Leng, Yifeng Zeng, Yixiao Lin, Ruimin Chen, Qifa Zhou, Andrea R. Hagemann, Lindsay M. Kuroki, Carolyn K. McCourt, David G. Mutch, Matthew A. Powell, Ian S. Hagemann, Quing Zhu

**Affiliations:** 1 Biomedical Engineering, Washington University, St Louis, MO 63130 USA; 2 Applied Bioptics LLC, St Louis, MO 63146 USA; 3 Department of Biomedical Engineering and Ophthalmology, University of Southern California, Los Angeles, CA 90089 USA; 4 Department of Obstetrics & Gynecology, Washington University School of Medicine, St. Louis, MO 63110 USA; 5 Department of Pathology & Immunology, Washington University School of Medicine, St. Louis, MO 63110 USA; 6 Department of Radiology, Washington University School of Medicine, St. Louis, MO 63110 USA

**Keywords:** Biophysics, Imaging and sensing

## Abstract

Ovarian cancer is the leading cause of death among gynecological cancers, but is poorly amenable to preoperative diagnosis. In this study, we investigate the feasibility of “optical biopsy,” using high-optical-resolution photoacoustic microscopy (OR-PAM) to quantify the microvasculature of ovarian and fallopian tube tissue. The technique is demonstrated using excised human ovary and fallopian tube specimens imaged immediately after surgery. Quantitative parameters are derived using Amira software. The parameters include three-dimensional vascular segment count, total volume and length, which are associated with tumor angiogenesis. Qualitative results of OR-PAM demonstrate that malignant ovarian tissue has larger and more tortuous blood vessels as well as smaller vessels of different sizes, while benign and normal ovarian tissue has smaller vessels of uniform size. Quantitative analysis shows that malignant ovaries have greater tumor vessel volume, length and number of segments, as compared with benign and normal ovaries. The vascular pattern of benign fallopian tube is different than that of benign ovarian tissue. Our initial results demonstrate the potential of OR-PAM as an imaging tool for fast assessment of ovarian tissue and fallopian tube and could avoid unnecessary surgery if the risk of the examined ovary is extremely low.

## Introduction

Ovarian cancer is the leading cause of death among gynecological cancers^1^. According to the American Cancer Society, about 22,530 patients will be diagnosed in 2019^2^, and 13,980 will die from ovarian cancer. Ovarian cancer is rarely diagnosed at early stage because of its low prevalence in the general population and the lack of effective screening techniques^3^. Strategies for ovarian cancer diagnosis include serum tumor marker CA-125 and imaging assessments^4,5^. Available imaging modalities, including ultrasonography, computed tomography, magnetic resonance imaging (MRI), and positron emission tomography, may confirm the presence of a pelvic mass. However, these imaging methods cannot reliably determine whether the mass is a cancer or a benign process. Under current clinical practice, women with a pelvic mass generally undergo salpingo-oophorectomy (SO)^6,7^. However, SO may result in morbidity and mortality, potentially including loss of fertility, premature menopause, accelerated bone loss and cardiovascular death^8^.

Laparoscopic, *in vivo* “optical biopsy” methods could potentially provide diagnostic results similar to conventional biopsy in real time and avoid unnecessary operations. Such optical biopsy methods include optical coherence tomography, which can capture changes in ovarian tissue light scattering due to collagen fiber erosion by cancer cells^9–11^; second-harmonic-generation microscopy, which can quantify the alteration of extracellular collagen matrix in malignant ovarian tissue^12^; and intraoperative fluorescence imaging^13^.

Photoacoustic tomography and microscopy are promising new techniques for cancer detection and diagnosis because of their enriched optical absorption contrasts, which are directly related to tumor vasculature and tumor oxygen saturation^14–18^. Transvaginal photoacoustic tomography provides data at conventional US resolution^17^, while high-resolution photoacoustic microscopy allows smaller features including microvascular morphology to be imaged^18^. Photoacoustic microscopy (PAM) can be divided into optical-resolution (OR) PAM and acoustic-resolution (AR) PAM. OR-PAM scans the focused light beam across the tissue surface and provides 3-D high-optical-resolution images revealing surface vasculature distribution, while AR-PAM scans the tissue with a focused high-frequency ultrasound transducer and provides acoustic-resolution images. AR-PAM can image more deeply than OR-PAM due to the use of acoustic focusing, but at the expense of resolution. Many research groups have explored OR-PAM in various clinical applications, such as human oral cavity^19^, skin^20^ and breast cancer^21^ imaging and diagnosis. This manuscript reports the first study of OR-PAM for imaging and quantification of microvascular components of human ovary and fallopian tube, which is suspected as the origin of high-grade serous carcinoma.

## Results

**Figure 1:**
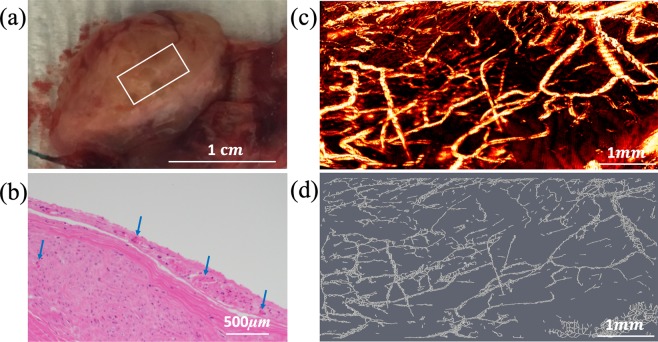
(**a**) photograph of a normal ovary from a 59-year-old post-menopausal woman. The white box marks the photoacoustic imaging area, (**b**) corresponding H&E image with blue arrows pointing to small blood vessels, (**c**) high-resolution OR-PAM MAP image of blood vessels, (**d**) spatial graph view of skeletonized vasculature with 2.35 normalized segments, 42.28 *mm*^3^ normalized total volume, and 34.16 *mm* normalized total length.

**Figure 2:**
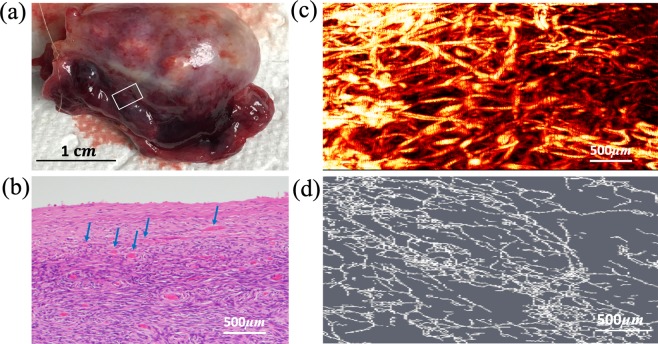
(**a**) photograph of an excised benign serous cystadenoma from an 87-year-old postmenopausal woman, white box identifying the imaging area, (**b**) corresponding H&E image with blue arrows highlighting blood vessels, (**c**) high-resolution OR-PAM MAP image of blood vessels, (**d**) spatial graph view of skeletonized vasculature with 2.92 normalized segments, 93.56 *mm*^3^ normalized total volume, and 55.11 *mm* normalized total length.

Eight benign ovaries, two malignant ovaries, and three benign fallopian tubes were included in the study (Table 1). We hypothesized that photoacoustic imaging properties of ovarian cancer would differ from those of benign ovaries. Figure 1 shows an example of a normal ovary from a 59-year-old post-menopausal woman. The OR-PAM imaging area of 3 mm by 6 mm is marked by the white box on the photograph (a). The corresponding H&E slide, the OR-PAM maximum amplitude projection (MAP) image, and the Amira skeletonized spatial graph view (see Methods) are given in (b)-(d), respectively. The MAP reveals many small blood vessels of a similar size and the Amira skeletonized map shows a more discretized vascular pattern. The H&E slide shows many smaller vessels on the surface. The three quantitative parameters from Amira are normalized segments 2.35, normalized total volume 42.28 *mm*3, and normalized total length 34.16 *mm*. Normalization allows comparisons between different imaging areas. Figure 2 shows a second example of a benign ovary with serous cystadenoma from an 87-year-old postmenopausal woman. The OR-PAM imaging area of 3 mm by 1.5 mm is marked by the white box on the photograph (a). The MAP (c) again reveals many smaller blood vessels of uniform size which can be seen well from the Amira skeletonized spatial graph view (d). The quantitative parameters are normalized segments 2.92, normalized total volume 93.56 *mm*^3^, and normalized total length 55.11 *mm*.

**Table 1:** Specimen characteristics (9 patients of 10 ovaries and 3 fallopian tubes, average age 54 years; range 42–64).

Cancerous ovaries	High-grade serous carcinoma (n = 2, average size 9.3 cm, range 8.5–10 cm)
Benign ovaries	Cystic follicles (n = 1, size 3 cm) Serous/mucinous cystadenoma (n = 3, average size 6.0 cm, range 5–7 cm) No significant histopathologic abnormalities (n = 4)
Normal/benign fallopian tubes	No significant histopathologic abnormalities (n = 3)

**Figure 3:**
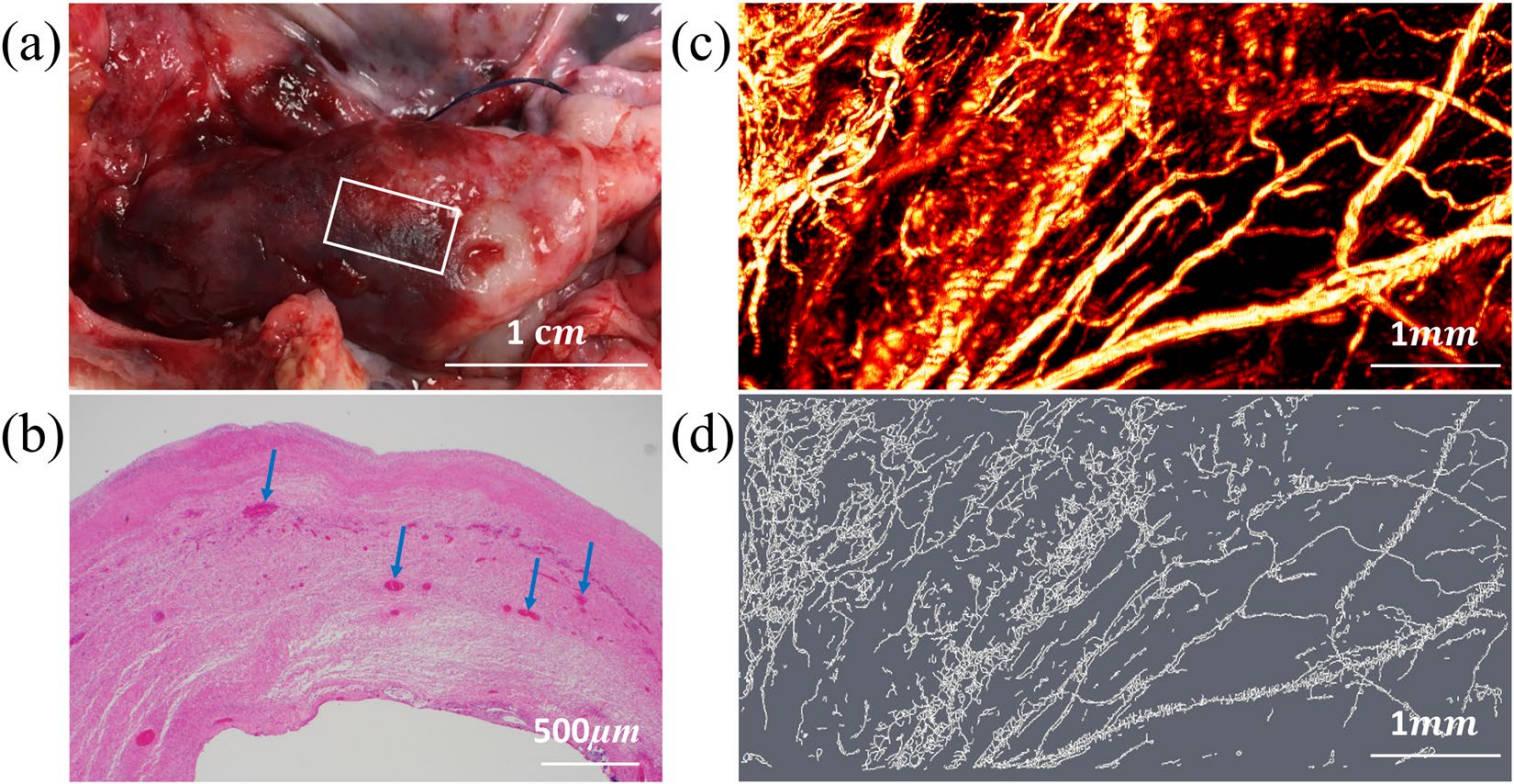
(**a**) photograph of an excised high grade carcinoma (mixed clear cell and endometrioid types) from a 61-year-old post-menopausal woman, white box identifying the imaging area, (**b**) corresponding H&E image with blue arrows highlighting larger blood vessels, (**c**) high-resolution OR-PAM MAP image of blood vessels, (**d**) spatial graph view of skeletonized vasculature with 4.78 normalized segments, 185.42 *mm*^3^ normalized total volume, and 82.61 *mm* normalized total length.

**Figure 4:**
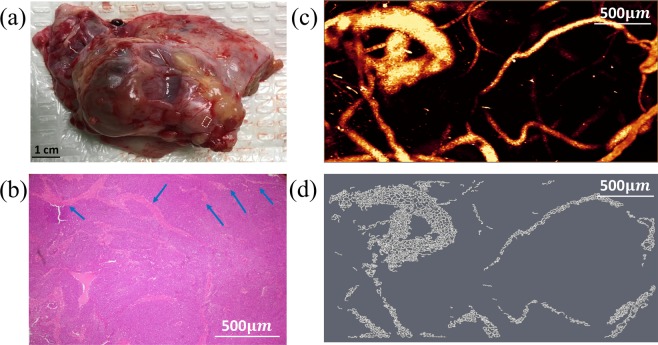
(**a**) photograph of an excised high-grade serous carcinoma from a 55-year-old premenopausal woman, white box identifying the imaging area, (**b**) corresponding H&E image with blue arrows highlighting larger blood vessels, (**c**) high-resolution OR-PAM MAP image of blood vessels, (**d**) spatial graph view of skeletonized vasculature with 5.95 normalized segments, 104.32 *mm*^3^ normalized total volume, and 43.83 *mm* normalized total length.

The vascular maps of malignant ovaries are dramatically different. Figure 3 shows an example of a high grade carcinoma with mixed clear cell and endometrioid from a 61-year-old post-menopausal woman. The OR-PAM image area of 3 mm by 6 mm is marked on the photograph (a). The OR-PAM MAP image reveals many thick larger vessels (right part of the image) as well as many smaller vessels of different sizes (c). The Amira skeletonized vasculature map helps visualize the different sizes of vessels, and the quantitative parameters show significant increase in the number of segments (4.78), the total volume (185.42 *mm*^3^), and the total length (82.61 mm) as compared with the normal and benign examples. Figure 4 shows a second example of a high-grade serous carcinoma from a 55-year-old premenopausal woman. The OR-PAM imaging area is 3 mm by 1.5 mm as marked by the white box on the photograph. Large and tortuous vessels can be seen from the OR-PAM MAP image and the quantitative parameters are normalized segments 5.95, normalized total volume 104.32 *mm*^3^, and normalized total length 43.83 *mm*. The segments and volume are about 2 times higher than that of the normal ovary.

**Figure 5:**
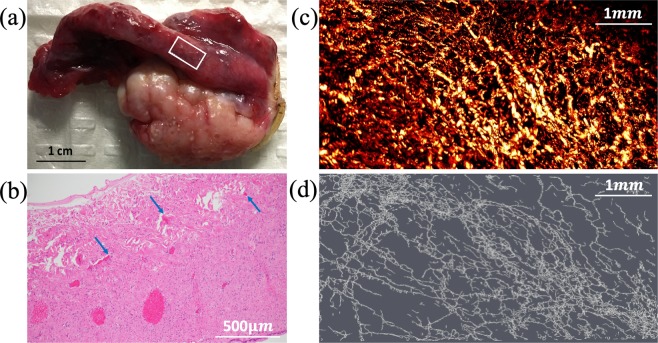
(**a**) photograph of a fallopian tube attached to an excised benign ovary, white box showing the imaging area, (**b**) H&E image of fallopian tube with blue arrows pointing to small vessels, (**c**) high-resolution OR-PAM MAP image of fallopian tube, (**d**) spatial graph view of skeletonized vasculature with 2.64 normalized segments, 61.66 *mm*^3^ normalized total volume, and 52.39 *mm* normalized total length.

Since high-grade serous ovarian cancer (the most common type of ovarian cancer) is believed to originate from fallopian tube epithelium, we anticipated that discriminating benign from malignant findings at the fimbriated end of the tube might become a clinically relevant task. To understand whether OR-PAM can be used for this task, we studied the properties of benign fallopian tubes attached to excised ovaries. An example of a normal fallopian tube from a 57-year-old postmenopausal woman is shown in Fig. 5. The OR-PAM image area is 3 mm by 6 mm. As seen from the OR-PAM MAP, a highly tortuous micro-vessel network with numerous branching small vessels is observed as shown in (c) and (d).

**Figure 6:**
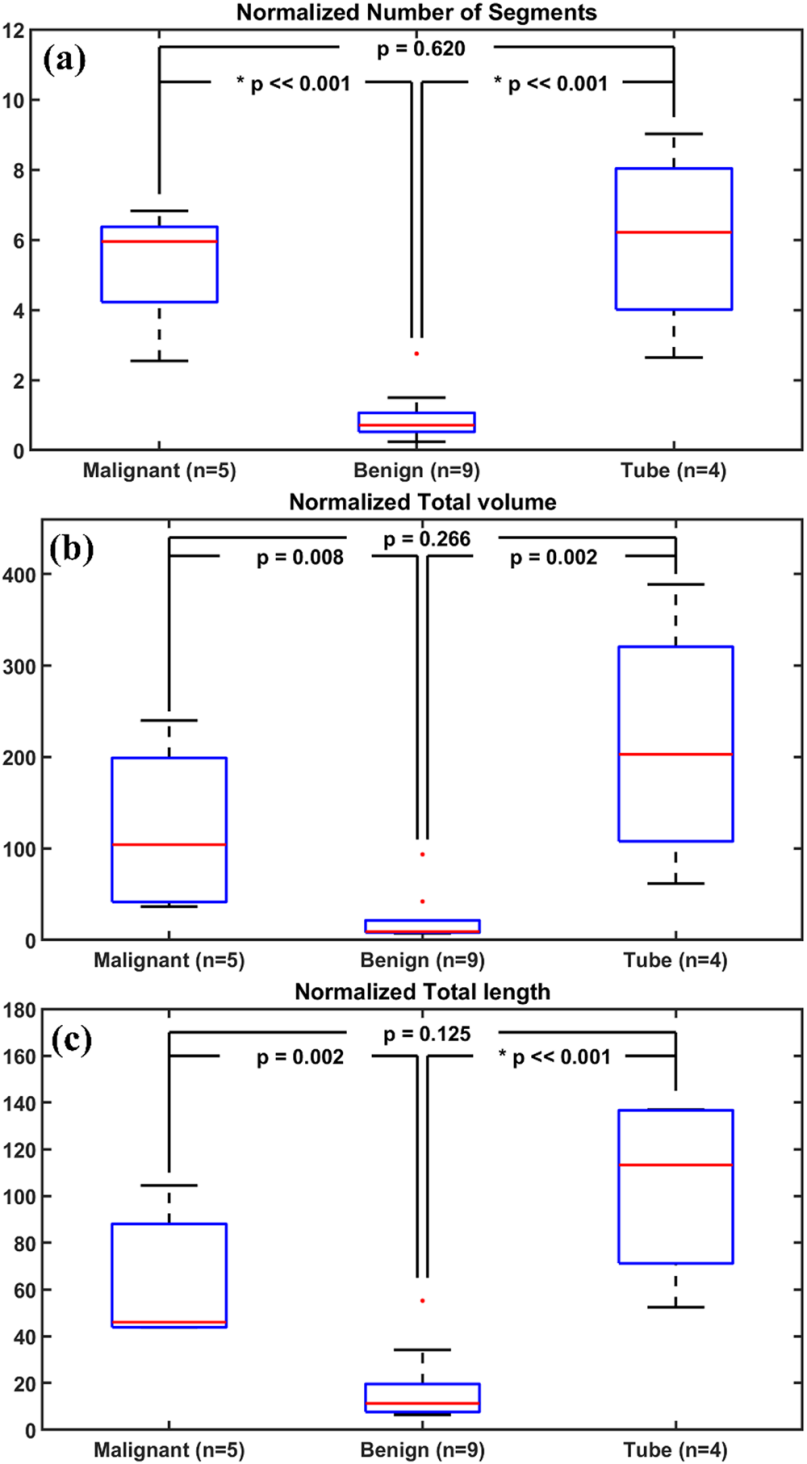
(**a**) Segment count, (**b**) total blood vessel volume in *mm*^3^, and (**c**) total blood vessel length in *mm* extracted from photoacoustic images of malignant ovary, benign ovary, and benign fallopian tube. n corresponds to the total number of imaged areas in each category.

Figure 6 shows the statistics of three quantitative parameters of normalized segment count, volume, and length obtained from three groups of malignant, normal/benign ovaries, and normal/benign fallopian tubes. All three parameters show statistically significant differences between malignant and normal/benign ovarian tissues. However, the range of the three parameters for the normal/benign fallopian tube group is much larger as compared with the malignant and normal/benign ovarian tissue groups. There is no statistical difference between the malignant ovaries and normal/benign fallopian tubes based on these vascular parameters, but the normal/benign ovarian tissue vascular parameters are statistically different than the normal/benign fallopian tubes.

## Discussion

In this study, we report the first qualitative and quantitative OR-PAM results of ovarian tissue and fallopian tube. Qualitatively, OR-PAM images were acquired by either a small or a large 3D scanning mode and then visualized by Amira. An auto-skeleton module was applied to all 3D scans and a spatial graph statistics module was used to extract quantitative features including number of segments, total volume, and total length. All these features are directly related to the level of tissue vasculature. Our OR-PAM results agree with the *in vivo* transvaginal photoacoustic imaging findings that invasive epithelial ovarian cancers showed more extensive tumor vascularity than benign and normal ovaries^17^.

Visually, there are differences in the microvascular networks of benign ovaries, malignant ovaries, and benign fallopian tubes. In the benign ovarian tissue, the microvasculature consists of small vessels of uniform size. Notably, the malignant ovary’s blood vessels show larger, twisted vessels and more branches as well as smaller vessels of different sizes. This is assumed to be the result of extensive tumor angiogenesis associated with malignancy. Since the auto-skeleton module in Amira not only counts the number of vessels, but also the thickness and the degree of distortion of vessels, the malignant ovarian tissue microvascular network shows more normalized segments, normalized total volume, and normalized total length as compared to the well-organized and more uniform microvessel network of benign and normal ovarian tissue. Unlike the benign ovaries, a highly tortuous microvessel network with numerous branching small vessels is observed in fallopian tubes. The fallopian tube is the origin of high-grade serous carcinoma, which is the most common and most lethal subtype of ovarian cancer^22–24^. Due to the lack of malignant fallopian tubes in this study, we cannot determine if there are differences between malignant and benign tubes. This is an interesting and important subject for a future study. Specifically, a larger dataset is planned in the future to include more ovarian tissues representing different diagnoses, so that an accurate classification model can be developed based on ovarian tumor microvessel network segments, total volume and total length for identifying malignant ovarian lesions.

The ovarian tissue H&Es are in a plane perpendicular to the ovarian surface, while the OR-PAM MAP images are 2-D projection images. While the H&Es are taken from regions that correspond to the area imaged by OR-PAM, these are not meant to be isomorphic to one another. The qualitative comparison of H&Es and MAPs demonstrates that there are larger and more tortuous blood vessels in the malignant ovarian tissue (hence crossing the plane of section more often) and smaller and more uniform blood vessels in the normal and benign ovarian tissue.

Amira is a powerful, multifaceted 2D-5D platform for visualizing, manipulating, and analyzing life science research data. It has already been demonstrated and adapted in CT and MRI researches for 3D visualization and quantification of human upper airways and mouse brains^25–27^. This report describes the first application of Amira to ovarian tissue vasculature. Currently, the post-processing time for quantification was around an hour using a Dell XPS 8700 (Windows 7, Intel(R) Core(TM) i7-4790 CPU @ 3.60 GHz, 8GB RAM, NVIDIA GeForce GT 720). However, we do not anticipate this as a limiting factor for future clinical applications since most of the calculations could be optimized for a GPU platform or other parallel computing platforms.

## Conclusion

We report the development and application of a novel high optical-resolution photoacoustic microscopy (OR-PAM) system for imaging microvascular components of ovarian tissue and fallopian tube. For the first time, quantitative parameters for characterizing microvascular components of ovarian tissue and fallopian tube are derived using the Amira software. The parameters include three-dimensional vascular segment count, total volume and length, which are associated with tumor angiogenesis. Qualitative results of OR-PAM demonstrate that malignant ovarian tissue has larger and tortuous blood vessels as well as smaller vessels of different sizes, while benign and normal ovarian tissue has smaller vessels of uniform size. Quantitative analysis shows that malignant ovaries have greater tumor vessel volume, length and number of segments, as compared with benign and normal ovaries. Our pilot study suggests that OR-PAM may provide a valuable tool to assist surgeons for near real-time diagnosis. The tool is amenable to implementation as a tool for minimally invasive (laparoscopic) surgery. A modification of the current photoacoustic imaging system to include oxygen saturation mapping capability would provide yet more functional parameters, in order to facilitate intraoperative decision-making and reduce unnecessary surgical excisions when the risk of cancer in the examined ovary is extremely low.

## Methods and Materials

### Photoacoustic imaging system and probe

**Figure 7:**
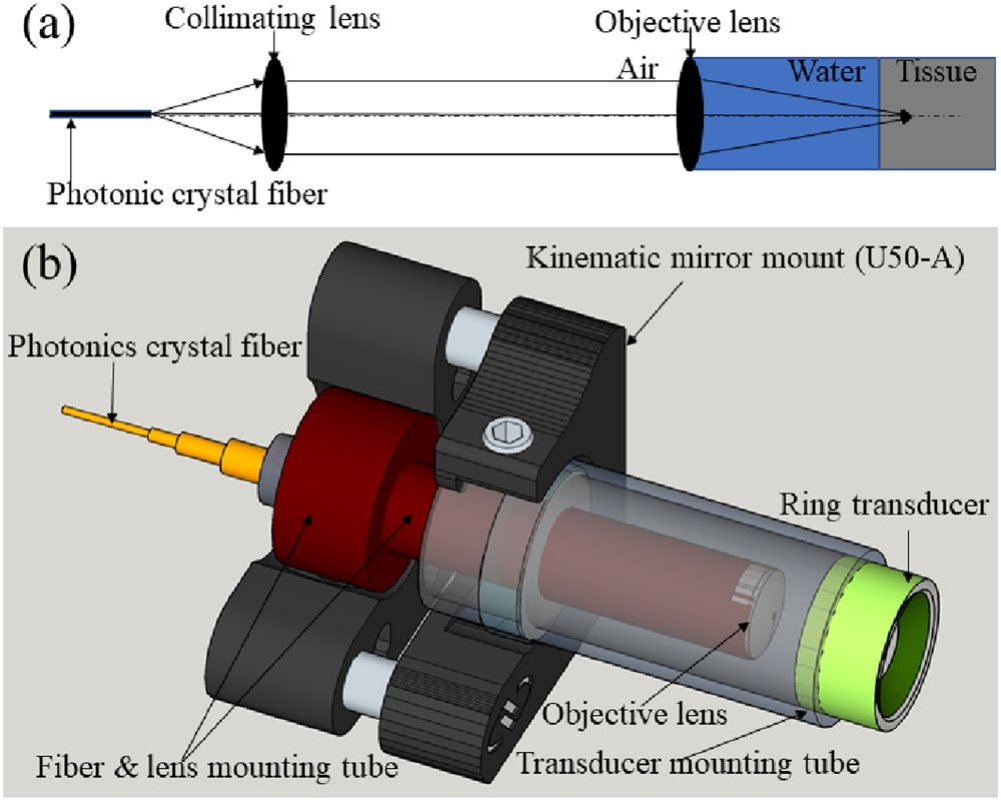
(**a**) schematic of the photoacoustic imaging system and (**b**) its imaging head.

A miniature photoacoustic imaging probe was constructed to integrate both laser beam delivering optics and a high-frequency ring transducer into a light assembly of less than 25 grams. It allows fast three-dimensional mechanical scanning with a first fast-x-scanning voice-coil stage and a second slow-y-scanning stage. 532 nm Nd: YAG laser with pulse repetition rate of 50 kHz was used in the study. A National Instrument analog output card was used to trigger the laser to obtain each A-scan line and each B-scan consisted of 1024 A-scans. Figure 7(a) illustrates the schematic of its optical layout where the pulsed 532 nm laser beam is delivered to the imaging probe via a photonics crystal fiber, collimated by an achromatic lens and focused by a water-immersed achromatic objective lens into tissue. As shown in Fig. 7(b), the imaging-probe assembly consists of a 1/2-inch diameter, clear quadrant mirror mount (U50-A), a fiber and lens mounting tube, a ring transducer mounting tube, a photonics crystal fiber patch cord, and a customized high-frequency (10–75 MHz) piezoelectric transducer with a center frequency of 40 MH, 6 dB bandwidth of 75% and focal depth of 6.35 mm. The fiber and lens mounting tube and the ring transducer mounting tube are secured to the mirror mount U50-A with a screw and epoxy separately. The confocal alignment of the laser beam focus and the ring transducer acoustic focus is achieved by adjusting the three hex-key actuators of U50-A. The achromatic objective lens is sealed at the end of the fiber and lens mounting tube. The entire imaging head shown in Fig. 7(b) is scanned together by a 2-axis motorized stage. During scanning, the relative position of transducer and optical component in imaging head is fixed. Water immersion of the back side of the achromatic objective lens is achieved by immerging the imaging probe in a water tank which is not shown in Fig. 7(b). The laser beam focus diameter was measured to be 6 µm by a CMOS camera-based beam profile device. The lateral resolution of the imaging probe was determined to be 3 µm. The signal-to-noise ratio of the imaging probe was quantified by imaging a graphite sample with a laser pulse energy of 5 nJ. After linearly scaling the laser pulse energy to 40 nJ without considering the non-linear effect, an averaged signal-to-noise ratio of 100 dB was estimated. Other integrated systems for performing OR-PAM have been reported^28,29^.

**Figure 8:**
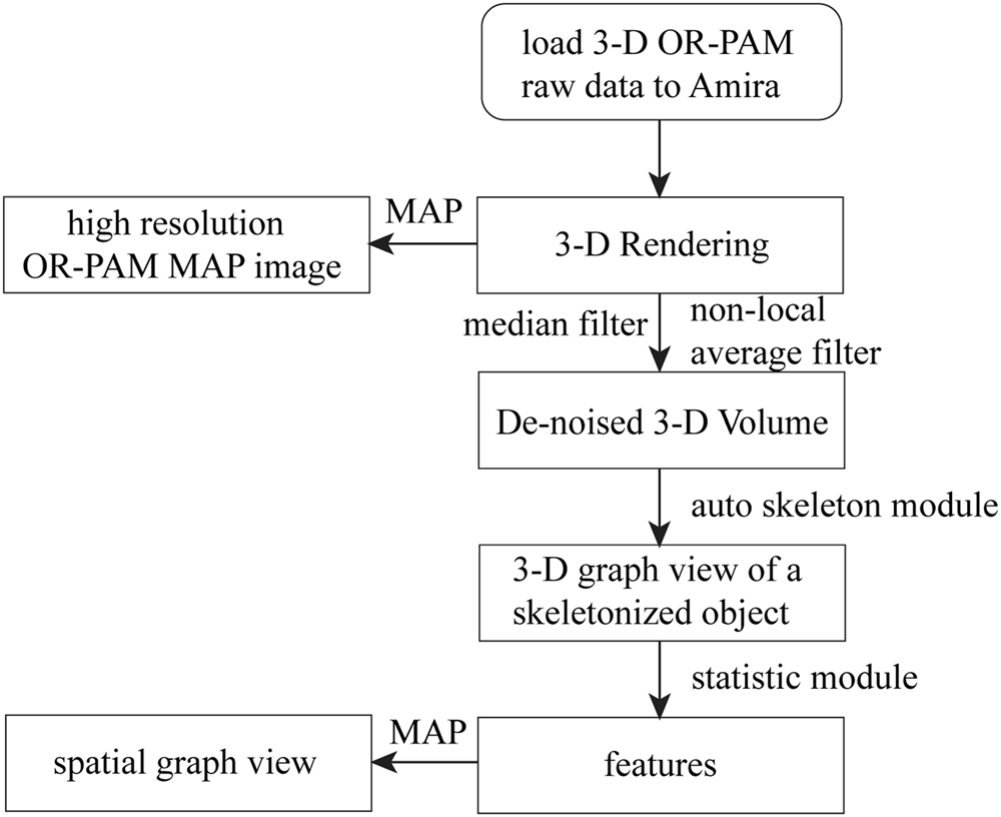
The post-processing flowchart using Amira 6.0.1.

A sinusoidal fast-scan waveform generated by a 12-bit analog output card with 2048 digital points and a 20 kHz output-clock rate is applied to a voice coil stage driver for each 3-mm-long B-scan image acquisition. A 2.5 µm separation between two neighbor B-scan images is controlled by the movement of a slow-axis step motor. 256 sampling points are acquired by an A/D card with 180 MHz sampling clock for each A-line image excited by each laser pulse. A customized multithread C++ program simultaneously performs the data acquisition, real-time front-end imaging display, real-time data streaming into computer memory by a background thread and post-processing raw data into a stack of B-scan images. In small 3D imaging mode, a 3 mm (fast x) by 1.5 mm (slow y) by 2.19 mm (depth) tissue volume is acquired with a total of 600 B-scan images within 1 minute and a binary raw data set of the 600 B-scan images is saved to a solid-state drive. In large 3D imaging mode, a 3 mm (fast x) by 6 mm (slow y) by 2.19 mm (depth) tissue volume is acquired with a total of 2400 B-scan images within 4 minutes and four binary raw data sets containing 2400 B-scan images are saved to the solid-state drive. Finally, a maximum-amplitude-projection image (MAP) is formed from all B-scan images after loading the data to Amira software (see Fig. 8). During imaging acquisition, the tissue surface laser intensity is maintained below the ANSI laser safety standard.

### Quantitative features extraction with Amira software

In post-processing mode, saved binary raw data are converted to B-scan images and analyzed quantitatively using a commercial 3D rendering and quantification software (Amira 6.0.1, Thermo Fisher Scientific, Waltham, MA)^30^. The post-processing flowchart is shown in Fig. 8. First, all OR-PAM B-Scan images generated from raw data acquired from the customized C++ program are loaded into Amira and the voxel setting in Amira is specified as 2.93 µm (x) by 8.56 µm (z) by 2.5 µm (y) volume. Then, a median filter with a window of 6 voxels is applied to enhance boundary definition and reduce background noise. Another non-local average filter is used to further reduce speckle noise. An auto-skeleton module is subsequently applied to divide the entire microvessel network in the image into many segments and find the centerlines of blood vessels. The module extracts the center line of filamentous structures from image data, which is segmented instantly with a user-defined threshold value. Specifically, it first calculates a distance map of the segmented image (Distance Map for Skeleton) based on the connectivity of signal intensity, and then performs a thinning of the label image using the Thinning module so that a final string of connected voxels remains (thinned), representing the centerline of the segment. The result is a 3-D graph view of a skeletonized object that can be visualized in the 3D viewer. Finally, a spatial graph statistics module is applied to extract quantitative features including number of segments, total volume, and total length from the 3-D data. The number of segments suggests how many branches exist within all blood vessels. The total volume and the total length reflect the size of the blood vessel networks. A projection image is generated from the 3-D graph view for visualization of the blood vessel network only and named as a spatial graph view. Note that all three features are normalized to the number of B-scans in each selected imaging area or volume. Student’s t-test is applied to these features to determine the statistical significance.

### Ovary sample preparation

This study was approved by the Institutional Review Board of Washington University School of Medicine, and informed consent was obtained from all patients. Due to the nature of the study, no patient was under 18 years old. Pathologists in the frozen section lab provided guidance to the researchers as to the sample orientation and location of the tumor, which was in any event not subtle. Therefore, we were certain that we correctly identified malignant and benign/normal areas for imaging. Specimens were imaged immediately after surgical resection and returned to the Pathology Department within an hour for routine processing. The image duration was around five minutes for each specimen. Each sample was imaged from one to three areas depending on the specimen size. Each imaged area is far enough from the others to maintain independence. Before each imaging study, the imaging probe was immersed in a water tank with a disposable thin plastic membrane bottom. Transparent ultrasound gel or water was applied between the tissue surface and the plastic membrane bottom for good acoustic contact. The plastic membrane was replaced, and the imaging probe was disinfected after each imaging study session in order to avoid cross-contamination between samples. All methods were performed in accordance with the relevant guidelines and regulations.

### Data analysis

Statistical analysis was performed using MATLAB R2016a. The student’s t-test was used to test the significance between groups and p < 0.05 was considered statistically significant. The vascular features were extracted from Amira 6.0.1.

## Data Availability

The data that support the plots within this paper and other findings of this study are available from the corresponding author upon request.
